# Calpain 4 Is Not Necessary for LFA-1-Mediated Function in CD4+ T Cells

**DOI:** 10.1371/journal.pone.0010513

**Published:** 2010-05-07

**Authors:** Sarah A. Wernimont, William T. N. Simonson, Peter A. Greer, Christine M. Seroogy, Anna Huttenlocher

**Affiliations:** 1 Program in Cellular and Molecular Biology, University of Wisconsin, Madison, Wisconsin, United States of America; 2 Department of Pathology and Molecular Medicine, Queen's University, Kingston, Ontario, Canada; 3 Departments of Pediatrics and Medical Microbiology and Immunology, University of Wisconsin, Madison, Wisconsin, United States of America; New York University, United States of America

## Abstract

**Background:**

T cell activation and immune synapse formation require the appropriate activation and clustering of the integrin, LFA-1. Previous work has reported that the calpain family of calcium-dependent proteases are important regulators of integrin activation and modulate T cell adhesion and migration. However, these studies have been limited by the use of calpain inhibitors, which have known off-target effects.

**Methodology/Principal Findings:**

Here, we used a LoxP/CRE system to specifically deplete calpain 4, a small regulatory calpain subunit required for expression and activity of ubiquitously expressed calpains 1 and 2, in CD4+ T cells. CD4+ and CD8+ T cells developed normally in Capn4^F/F^:CD4-CRE mice and had severely diminished expression of Calpain 1 and 2, diminished talin proteolysis and impaired casein degradation. Calpain 4-deficient T cells showed no difference in adhesion or migration on the LFA-1 ligand ICAM-1 compared to control T cells. Moreover, there was no impairment in conjugation between Capn4^F/F^:CD4-CRE T cells and antigen presenting cells, and the conjugates were still capable of polarizing LFA-1, PKC-theta and actin to the immune synapse. Furthermore, T cells from Capn4^F/F^:CD4-CRE mice showed normal proliferation in response to either anti-CD3/CD28 coated beads or cognate antigen-loaded splenocytes. Finally, there were no differences in the rates of apoptosis following extrinsic and intrinsic apoptotic stimuli.

**Conclusion/Significance:**

Our findings demonstrate that calpain 4 is not necessary for LFA-1-mediated adhesion, conjugation or migration. These results challenge previous reports that implicate a central role for calpains in the regulation of T cell LFA-1 function.

## Introduction

CD4+ T cells are important regulators of the adaptive immune response. Following T cell receptor (TCR) recognition of its cognate peptide-MHC complex and costimulation, signaling leads to prolonged T cell:APC interactions, described as an immune synapse, which culminates in T cell activation, proliferation and cytokine production [1]. Formation of the immune synapse requires the activity of the integrin LFA-1 (αLβ2), and mice lacking LFA-1 exhibit impaired T cell proliferation and cytokine responses following antigen exposure in vivo [2].

LFA-1 activity is regulated both by an upregulation of affinity for its ligand and clustering at the immune synapse following TCR stimulation (reviewed in [3]). The cytoskeletal regulatory protein, talin, is an important positive regulator of both LFA-1 affinity and clustering in T cells. Binding of the talin FERM domain containing head region to the cytoplasmic tail of LFA-1 can increase integrin affinity for ligand and association of the talin rod domain can promote integrin clustering [4]. Previous work has shown that the calpain family of cysteine proteases can cleave talin between the head and rod domain, thereby modulating the ability of talin to activate integrins [5]. Thus, there has been considerable speculation and studies investigating the possible roles of calpain in T cell integrin regulation.

The calpain family of cysteine proteases have been found to have an important role in a diverse number of cellular events ranging from regulation of the actin cytoskeleton (reviewed in [6]) to modulation of apoptosis [7]. There are two ubiquitously expressed isoforms of calpain, calpain 1 (u) and calpain 2 (m), that are distinguished by their calcium requirements for activity. These isoforms form heterodimers with the small calpain subunit, calpain 4, which stabilizes calpain 1 and 2. In the absence of calpain 4, there is a loss of both calpain 1 and 2 expression and activity [8]. Knockout of calpain 4 is embryonic lethal [8], and fibroblasts obtained from calpain 4 knockout mice have been shown to have impaired migration [9] and altered responses to apoptotic stimuli [7].

Given the known importance of integrins for T cell activation, there has been considerable interest in dissecting the role that calpains may play in T cell adhesion and migration. To date, the function of calpains in T cells has been studied using cell permeable inhibitors. Several reports using calpain inhibitors have shown that calpain is required for T cell adhesion mediated by β1 and β2 integrins [10], [11], [12], [13]. However, a more recent study has suggested calpain is not required for T cell adhesion to ICAM-1 and that some of the initial findings of decreased adhesion may have been due to off target effects of inhibitors and rapid induction of apoptosis following stimulation [14].

No study has yet investigated the role of calpain in T cell biology using targeted calpain depletion. Here, we use the LoxP-CRE system to specifically disrupt calpain 4 expression in CD4+ T cells. T cells develop normally in these mice and were used to study T cell migration, adhesion, conjugation, proliferation and apoptosis. Surprisingly, we found no impairment in any of these functions in calpain 4-deficient CD4+ T cells.

## Results

### Calpain 4 is not required for T cell development

**Figure 1:**
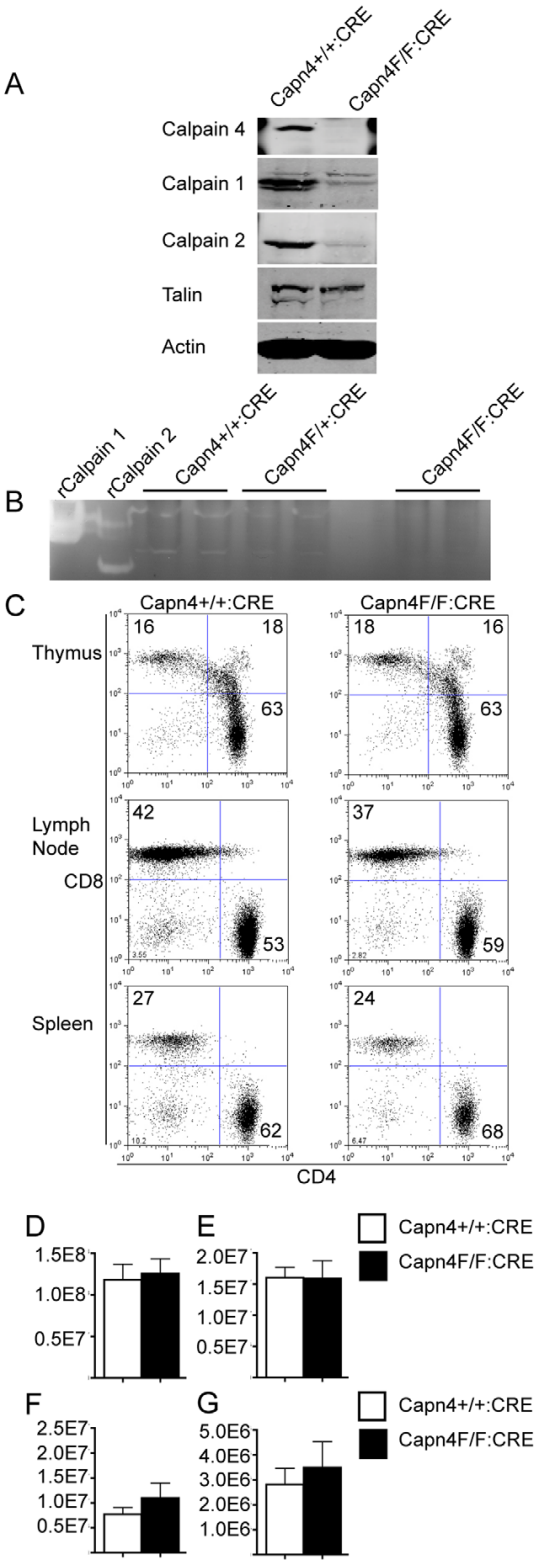
Calpain 4 is not required for T cell Development. A) Immunoblot of anti-CD3/CD28 bead activated CD4+ T cells from Capn4^+/+^:CD4-CRE and Capn4^F/F^:CD4-CRE mice probed with antibodies to calpains 1, 2 and 4, talin and actin. Capn4^F/F^:CD4-CRE cells have no calpain 4 expression and significant decreases in calpain 1 and 2 expression. Notably, there is loss of talin proteolysis in Capn4^F/F^:CD4-CRE lysates, indicating loss of calpain activity in these cells. B) Casein zymography, which detects the ability of calpains to degrade a known substrate, casein, shows calpain activity in anti-CD3/CD28 bead activated CD4+ T cells from Capn4^+/+^:CD4-CRE, Capn4^F/+^:CD4-CRE but not Capn4^F/F^:CD4-CRE mice. CD4+ T cells were lysed and run on a casein-acrylamide gel under non-reducing conditions. Reactivation of calpains resulted in casein degradation. Gel was stained by coomassie, and casein degradation is seen as area of clearing. C) Thymus, lymph nodes and spleen of Capn4^+/+^:CD4-CRE and Capn4^F/F^:CD4-CRE mice were pulverized and cells stained with anti CD3, CD4 and CD8 antibodies. Thymus staining is from 6 week old mice. Lymph node and spleen staining is from 5 month old mice. Graphs are average cell numbers +/− SEM from 4 wild type and 6 knockout mice at 5 months of age: D) Total number of splenocytes E) Total number of cells in 2 inguinal and 2 cervical lymph nodes F) Number of CD4+ T cells in spleen G) Number of CD4+ T cells in lymph nodes.

Genetic depletion of the calpain 4 small subunit in mice is embryonic lethal [8]. In order to test the role of calpain 4 in T cells, floxed calpain 4 mice [15], with loxP sites flanking exons 9 and 11, were crossed with mice expressing CRE recombinase driven off the CD4 promoter. CD4+ T cells from these Capn4^F/F^:CD4-CRE mice did not express calpain 4, consistent with previous findings, and showed a significant decrease in calpain 1 and 2 expression compared to Capn4^+/+^:CD4-CRE control cells (Figure 1A). We detected no calpain activity in Capn4^F/F^:CD4-CRE CD4+ T cells using casein zymography, which detects calpain activity in cell lysates as an area of clearing in an acrylamide gel supplemented with the calpain substrate, casein (Figure 1B). These findings were similar to those reported with calpain 4−/− fibroblasts [9]. Additionally, we observed a significant decrease in talin cleavage suggesting that calpain activity is impaired in calpain 4-deficient T cells (Figure 1A).

T cells from Capn4^F/F^:CD4-CRE mice developed normally, and we observed no change in the CD4 and CD8 distribution in thymic populations of 6 week old Capn4^F/F^:CD4-CRE mice compared to control mice (Figure 1C). While there was a trend toward increased CD4+ T cells in Capn4^F/F^:CD4-CRE mice compared to Capn4^+/+^:CD4-CRE mice, there was no statistically significant difference in the total number of CD4+ T cells found in lymph nodes and splenic fractions between wild type and knockout mice at 5 months of age (Figure 1 C–G). Together, these results suggest that calpain 4 is not required for T cell development or homeostasis.

### Calpain 4 is not required for CD4+ T cell migration

Calpains have been identified as both positive and negative regulators of cell migration (reviewed in [6]). Calpain 4-deficient fibroblasts have impaired migration [9]. In contrast, calpain is a negative regulator of neutrophil and monocyte chemokinesis [16], [17]. The role of calpains in lymphocyte migration is more controversial: one recent study reported that calpain is required for Jurkat T cell and human T cell chemotaxis [18] whereas another study showed no role for calpain in lymphocyte migration [17].

**Figure 2:**
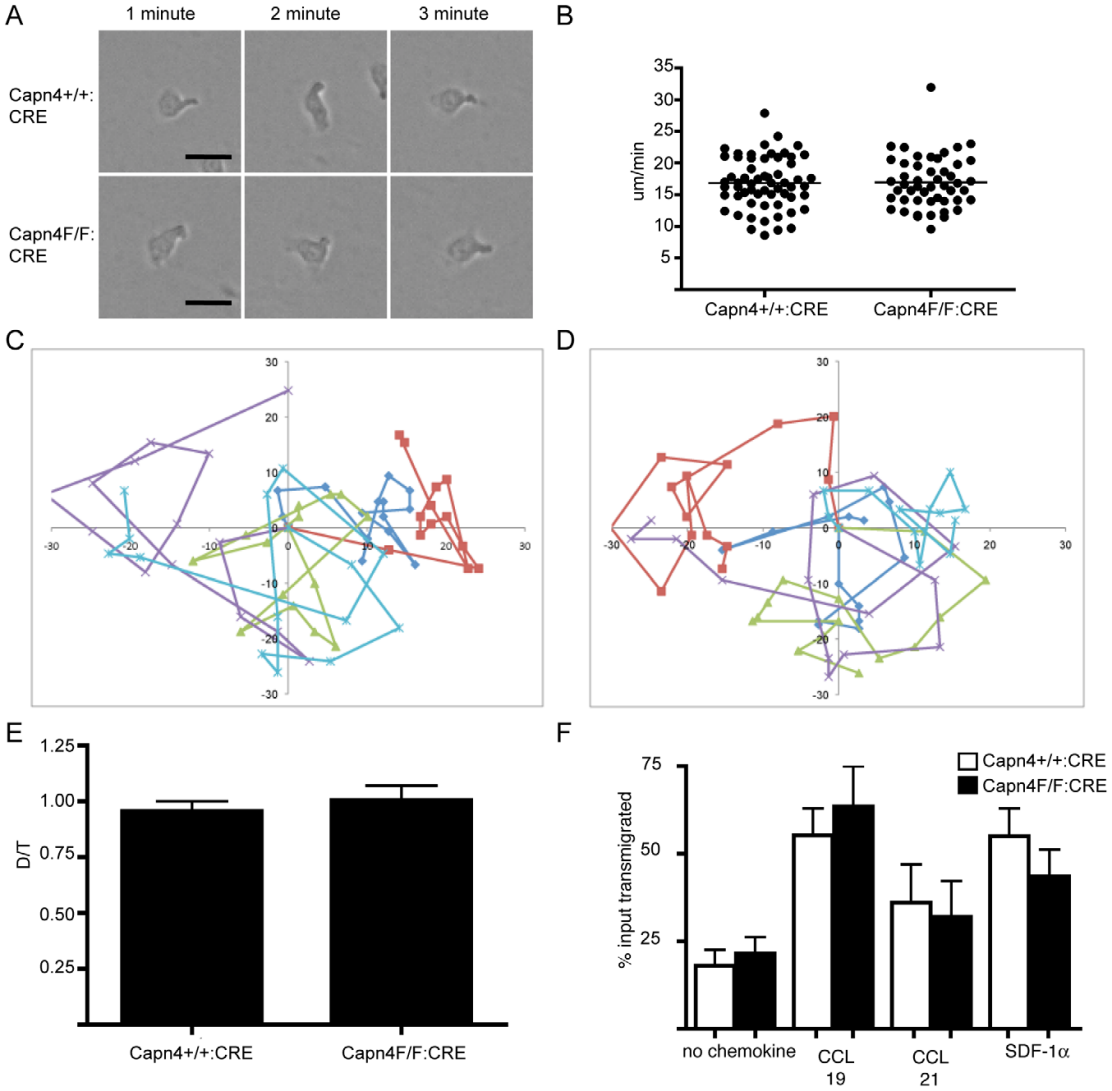
Calpain 4 is not required for CD4+ T cell random migration on ICAM-1 or chemotaxis. A) Representative images of Capn4^+/+^:CD4-CRE and Capn4^F/F^:CD4-CRE anti-CD3/CD28 bead activated CD4+ T cells migrating on mICAM-1. Scale bar represents 10 um. B) Anti-CD3/CD28 bead activated CD4+ T cells from Capn4^+/+^:CD4-CRE and Capn4^F/F^:CD4-CRE mice were imaged while migrating on mICAM-1. Cells were tracked and velocity determined using ImageJ. Values are from 3 independent experiments +/− SEM. C) Cell tracks of 5 representative Capn4^+/+^:CD4-CRE cells D) Cell tracks of 5 representative Capn4^F/F^:CD4-CRE cells E) Directionality of Capn4^+/+^:CD4-CRE and Capn4^F/F^:CD4-CRE cells measured as the ratio of net distance traveled (D) divided by total path length (T). F) Transwell migration of Capn4^+/+^:CD4-CRE and Capn4^F/F^:CD4-CRE CD4+ T cells was measured using 3 um transwells coated with fibronectin. Anti-CD3/CD28 bead activated CD4+ T cells were added to the upper transwell chamber and were allowed to migrate toward the lower chamber in the absence or presence of 100 ng/ml of CCL19, CCL21 or SDF-1 alpha. After 6 hours, the percentage of cells transmigrated to the lower chamber were quantified by flow cytometry. There is no difference in rates of transwell migration between Capn4^+/+^:CD4-CRE and Capn4^F/F^:CD4-CRE cells. Values are mean +/− SEM of 4 independent experiments.

**Figure 3:**
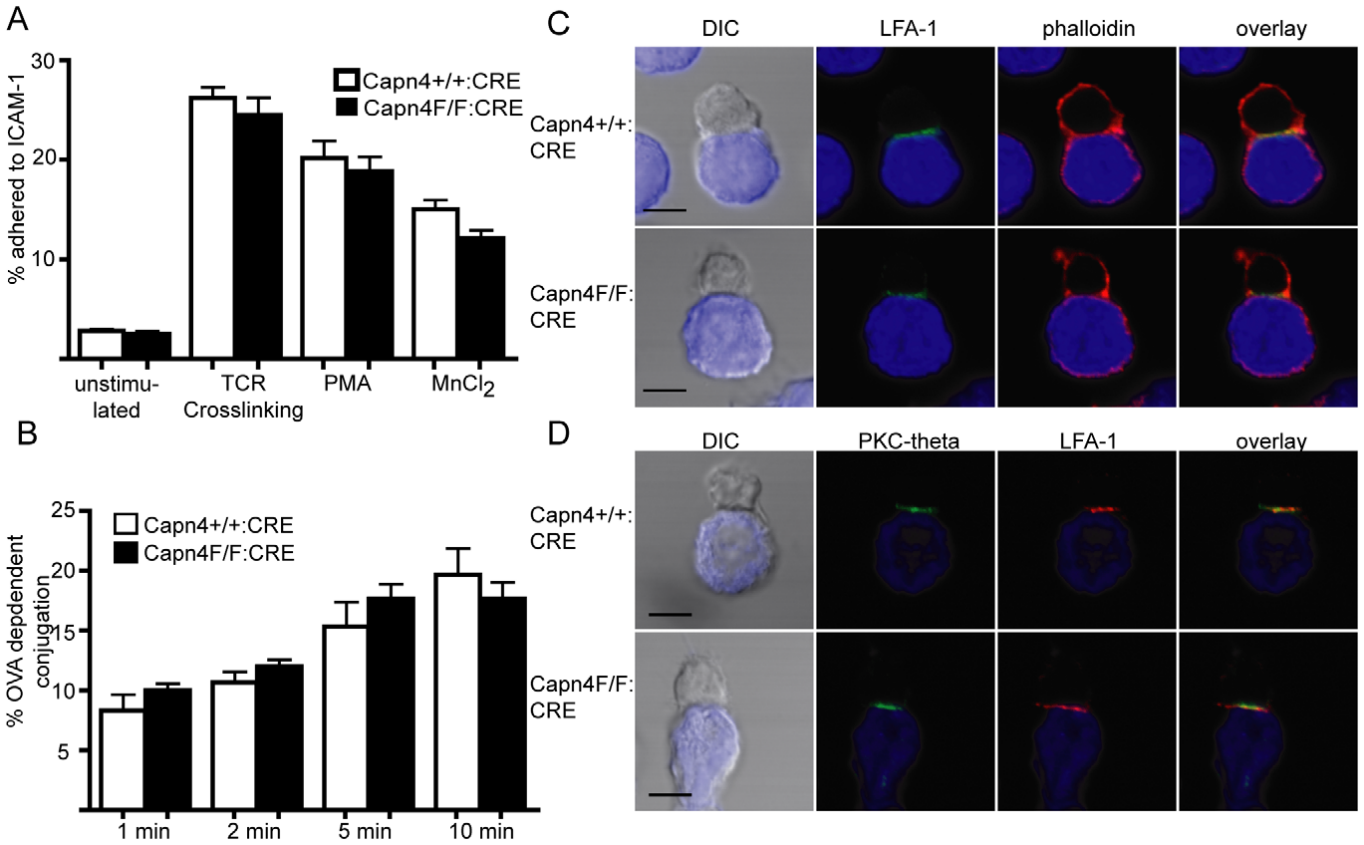
Calpain 4 is not required for CD4+ T cell adhesion, conjugation and Immune Synapse Formation. A) Anti-CD3/CD28 bead activated CD4+ T cells from Capn4^+/+^:CD4-CRE and Capn4^F/F^:CD4-CRE mice were fluorescently labeled and left unstimulated or stimulated with either CD3 crosslinking, PMA or MnCl2. Cells were allowed to adhere to mICAM-1-coated plates at 37C for 25 minutes, and adhesion was quantified by fluorescent plate reader before and after washing. Values are mean +/− SEM of 3 independent experiments B) OVApeptide expanded CD4+ T cells from Capn4^+/+^:CD4-CRE and Capn4^F/F^:CD4-CRE mice expressing the OTII transgenic T cell receptor were incubated with LB27.4 B cells +/− OVApeptide. Following incubation, cells were briefly vortexed and conjugates measured by flow cytometry. Values represent OVA dependent conjugation from three independent experiments +/− SEM. C/D) OVApeptide expanded Capn4^+/+^:CD4-CRE and Capn4^F/F^:CD4-CRE CD4+ T cells were combined with peptide bearing LB27.4 B cells (blue) and incubated for 20 minutes prior to fixing on poly-l-lysine coated coverslips. Cells were stained with antibodies against LFA-1 and PKC-theta and rhodamine phalloidin and visualized by confocal microscopy. Images are representative of 3 independent experiments and show normal LFA-1, actin and PKC-theta polarization in the absence of calpain 4. Scale bar represents 5 um.

To further investigate this question, we used time-lapse microscopy to observe CD4+ T cell migration from Capn4^+/+^:CD4-CRE and Capn4^F/F^:CD4-CRE on ICAM-1. Both Capn4^+/+^:CD4-CRE and Capn4^F/F^:CD4-CRE cells achieved a polarized morphology with a front edge pseudopod and a rear localized uropod on ICAM-1 (Figure 2A) and migrated randomly at approximately 16 um/minute (Figure 2B). We observed no differences in directionality between control and calpain 4-deficient T cells (Figure 3 C–E).

Additionally, we wanted to know if there were differences in the ability of calpain 4-deficient T cells to chemotax to chemokines CCL19, CCL21 and SDF-1 alpha. Using a transwell migration assay, we found no difference in the percentage of Capn4^+/+^:CD4-CRE and Capn4^F/F^:CD4-CRE CD4+ T cells migrating toward chemotactic stimuli (Figure 2F). Together, these findings suggest that calpain 4 is not required for T cell chemokinesis or chemotaxis.

### Calpain 4 is not required for CD4+ T cell adhesion, conjugation or immune synapse formation

Previous studies have suggested that calpain is required for T cell adhesion to ICAM-1 [10], [11] and efficient LFA-1 clustering [12] following T cell stimulation. To further address this question, we tested the ability of Capn4^+/+^:CD4-CRE and Capn4^F/F^:CD4-CRE cells to adhere to ICAM-1-coated surfaces. We found no difference in rates of basal adhesion in unstimulated cells. Additionally, following TCR cross-linking, PMA and MnCl2 treatment, there were no differences in the percentage of ICAM-1 adhesion between Capn4^+/+^:CD4-CRE and Capn4^F/F^:CD4-CRE CD4+ T cells (Figure 3A). The findings indicate that calpain 4 is not required for T cell adhesion to ICAM-1.

Appropriate LFA-1 activation is also required for efficient T cell:APC immune synapse formation and calpains have been identified as regulators of this process [19]. To test whether calpain 4 is required for T cell:APC conjugation, we crossed Capn4^+/+^:CD4-CRE and Capn4^F/F^:CD4-CRE mice onto the OTII transgenic TCR background which expresses a TCR recognizing OVA_223–230_. CD4+ T cells from these mice were isolated and allowed to interact with LB27.4 B cells with and without OVA peptide. Using a flow cytometry based conjugation assay, we found no difference in OVA-dependent conjugation following 1, 2, 5 or 10 minutes of interaction (Figure 3B). Taken together, our findings suggest that calpain 4 is not required for T cell adhesion or T cell:APC conjugation.

Immune synapse formation correlates with polarization of LFA-1, the actin cytoskeleton and signaling molecules such as PKC-theta to the T cell:APC interface [1]. Using laser scanning confocal microscopy, we found no difference in polarization of LFA-1, actin or PKC-theta to the immune synapse between Capn4^+/+^:CD4-CRE and Capn4^F/F^:CD4-CRE and antigen loaded LB27.4 cells, suggesting that calpain is not required for immune synapse formation (Figure 3 C,D).

### Calpain 4 is not required for CD4+ T cell proliferation

**Figure 4:**
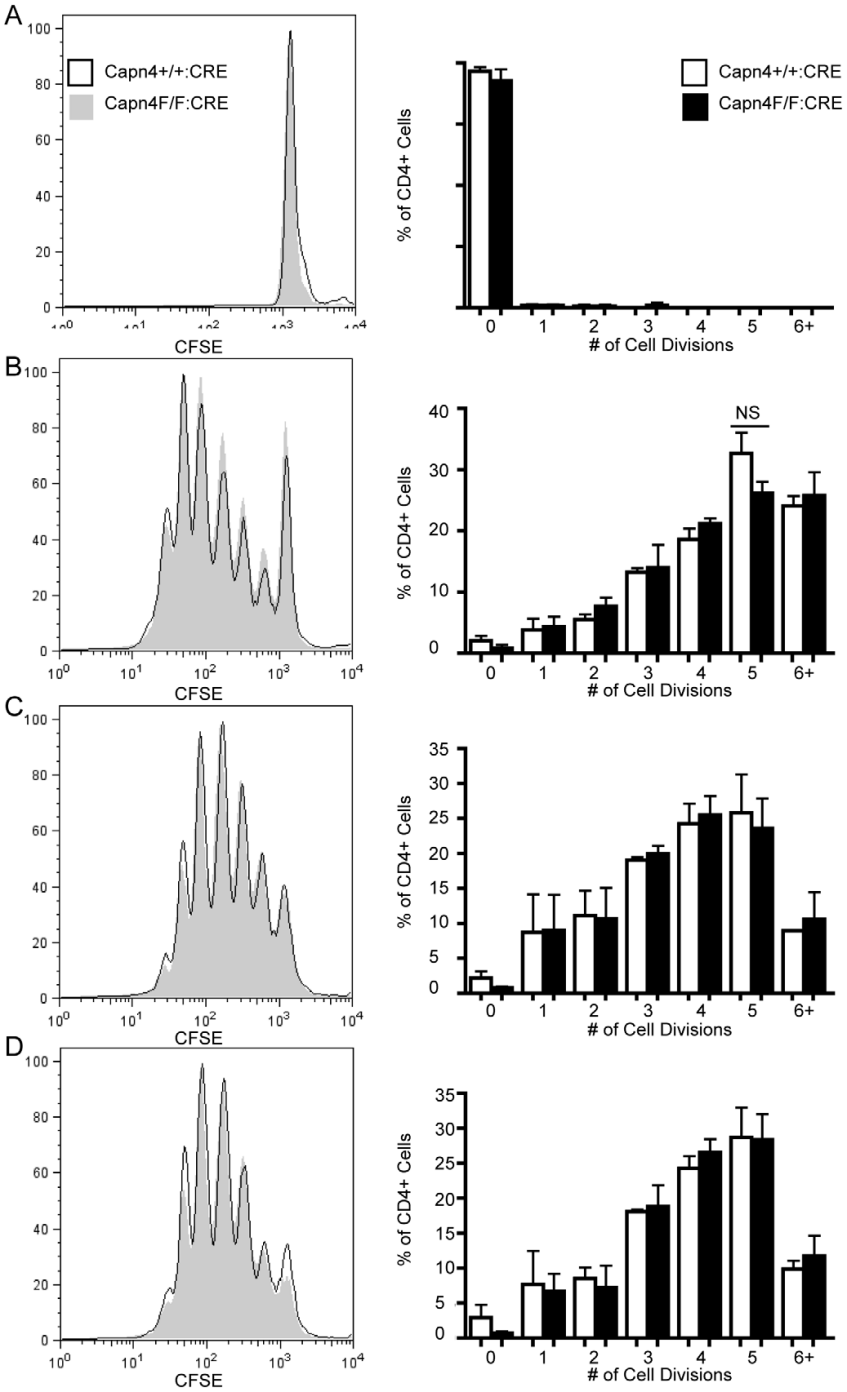
Calpain 4 is not required for CD4+ T cell proliferation. OVApeptide expanded CD4+ T cells from Capn4^+/+^:CD4-CRE and Capn4^F/F^:CD4-CRE mice were stained with CFSE 10 days following isolation. Cells were left unstimulated (A) or stimulated with anti-CD3/CD28 coated beads (B) or 0 (not shown), 0.1 ug/ml OVAp (C), 1 ug/ml OVAp (D). Following 3 days of stimulation, CFSE dilution was measured by flow cytometry. Plots are representative of 4 independent experiments and graphs are compilation of 4 independent experiments +/− SEM.

Consistent with its important role in mediating T cell:APC interactions, LFA-1 is also required for subsequent T cell activation and proliferation [2]. Given previous studies suggesting a role for calpain in LFA-1 regulation, we were interested in whether calpain 4 was required for efficient CD4+ T cell proliferation. Capn4^+/+^:CD4-CRE and Capn4^F/F^:CD4-CRE CD4+ T cells from OTII+ mice were stained with CFSE and left unstimulated or stimulated with anti-CD3/28 coated beads, 0.1 ug/ml OVApeptide or 1 ug/ml OVA peptide plus irradiated splenocytes for 72 hours (Figure 4). The degree of dye dilution corresponds to the degree of proliferation. We found that there was no difference in proliferation between Capn4^+/+^:CD4-CRE and Capn4^F/F^:CD4-CRE CD4+ cells following any of the stimulations, suggesting that calpain 4 is not required for T cell proliferation.

### Calpain 4 is not required for CD4+ T cell apoptosis

T cell homeostasis requires appropriate contraction of activated T cell populations following antigen-induced expansion. This is achieved in part by regulated apoptosis of T cells (Reviewed in [20]). Calpains have been identified as important regulators of caspase-dependent apoptosis. Fibroblasts from calpain 4 knockout mice have increased apoptosis in response to extrinsic signals and decreased apoptosis in response to intrinsic apoptotic stimuli [7]. Studies using calpain inhibitors in T cells have also supported a role for calpains in activation induced cell death [21], [22], apoptosis following etoposide induced DNA damage [23] and thymocyte apoptosis following glucocorticoid treatment [24].

**Figure 5:**
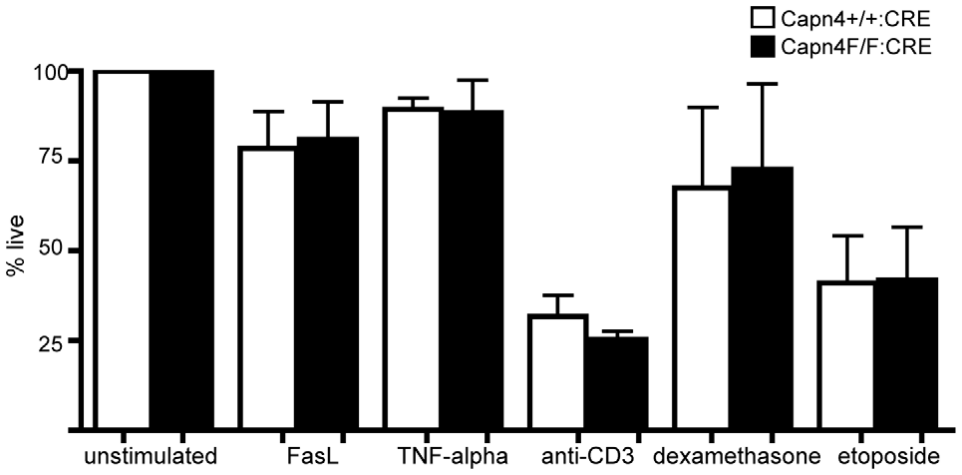
Calpain 4 is not required for apoptosis. CD4+ T cells from Capn4^+/+^:CD4-CRE and Capn4^F/F^:CD4-CRE mice were isolated and stimulated with anti-CD3/CD28 coated beads. Four days later, beads were removed and T cells were left unstimulated or stimulated with crosslinked FasL, TNF, crosslinked anti-CD3, dexamethasone or etoposide. 30 hours later, the percentage of apoptotic cells was determined by annexinV and propidium iodine staining using flow cytometry. Values are normalized to unstimulated controls and represent the mean +/− SEM from three independent experiments and show that calpain 4 is not necessary for induction of T cell apoptosis.

To determine if calpain 4 regulates T cell apoptosis, we treated CD4+ T cells from Capn4^+/+^:CD4-CRE and Capn4^F/F^:CD4-CRE with extrinsic and intrinsic apoptotic stimuli and monitored for early signs of apoptosis and cell death by annexin V and propidium iodide staining using flow cytometry. Following stimulation with crosslinked FasL and TNFα, we saw no differences in rates of cell death between Capn4^+/+^:CD4-CRE and Capn4^F/F^:CD4-CRE CD4+ T cells (Figure 5). Intrinsic pathway stimulation included treatment with the DNA topoisomerase inhibitor etoposide, the glucocorticoid dexamethasone and TCR crosslinking in the absence of costimulation. Again, we found no difference in rates of apoptosis between Capn4^+/+^:CD4-CRE and Capn4^F/F^:CD4-CRE CD4+ T cells with these stimuli (Figure 5). Together, these findings suggest that calpain 4 does not regulate T cell apoptosis induced by extrinsic or intrinsic stimuli.

## Discussion

Here, we provide the first report of genetic disruption of calpain 4 in T cells. Contrary to previous results using calpain inhibition of T cells, we found no defects in LFA-1 mediated adhesion, migration, immune synapse formation or proliferation. Likely, discrepancies between our findings and prior studies can be attributed to off target inhibitor effects since frequently used inhibitors ALLN, ALLM and calpastatin have all been reported to inhibit several other proteases and/or the proteosome [25], [26], [27], [28].

Regulation of LFA-1 through both affinity and avidity modulation is important for appropriate integrin activation in T cells (reviewed in [3]). Calpain has been an attractive candidate to regulate LFA-1 affinity in T cells since it is activated in response to calcium signaling which follows TCR ligation and it has been shown to cleave talin between its head and rod domain [5], which can potentially induce high affinity LFA-1 [29]. Additionally, clustering of LFA-1 has been shown to be dependent on release of LFA-1 from the actin cytoskeleton, since cytochalasin D treatment results in increased lateral mobility of LFA-1 in resting cells [12]. Calpain is an attractive candidate to initiate this cytoskeletal release since cleavage of talin may disrupt the linkage between LFA-1 and actin. Indeed, inhibition of calpain prevents stimulation-dependent decreases in lateral mobility in high affinity LFA-1 [12]

Recent work, however, has suggested that there is no change in talin cleavage following T cell stimulation suggesting that LFA-1 activation and clustering is not dependent on calpain-mediated proteolysis of talin [14]. Additionally, work from our lab has indicated that intact talin is required to induce LFA-1 clustering, and that the head domain alone cannot rescue LFA-1 clustering and support immune synapse formation [4]. In fibroblasts, we have found that talin proteolysis regulates focal adhesion disassembly [5]. Future investigations will determine whether or not calpains may modulate LFA-1 deactivation and disassembly in T cells.

Our findings demonstrate that LFA-1 activation following T cell stimulation is independent of calpain activity. We found no detectable impairment of adhesion, conjugation, LFA-1 polarization to the immune synapse or proliferation in calpain 4-deficient T cells. One possible criticism of this investigation may be that other isoforms of calpain could compensate for loss of calpain 4 activity and expression of calpain 1 and 2. However, talin proteolysis and the ability of calpain 1 and 2 to degrade casein was impaired in calpain 4-deficient T cells, indicating reduced calpain activity. Thus, we doubt that there is compensation by other calpains specifically fulfilling the functions of calpain 4 in these cells. However, it is possible that residual amounts of calpain 1 and 2, which remain following calpain 4 depletion, are still catalytically active even though we cannot detect their activity.

In summary, based on our findings, we think that the role of calpains in T cell LFA-1 function needs to be reconsidered. Moreover, future studies investigating calpain function should rely less on the use of inhibitors, which have known off-target effects, and center more on specific siRNA or genetic depletion of calpains.

## Materials and Methods

### Ethics Statement

We received specific approval for this study from the University of Wisconsin School of Medicine and Public Health Institutional Animal Care and Use Committee (protocol number MO1570-0-06-07).

### Mice

Calpain 4 flox/flox mice were generated as previously described [15]. Mice were backcrossed for at least 6 generations onto a C57/Black6 background and crossed with mice expressing CRE recombinase from the CD4 promoter (Taconic). Additionally, Capn4^+/+^:CD4-CRE and Capn4^F/F^:CD4-CRE mice were crossed with mice expressing the OTII transgenic TCR (Jackson Labs). The following genotyping primers were used for identification of Capn4^+/+^:CD4-CRE and Capn4^F/F^:CD4-CRE mice: Calpain 4: forward 5′ GTG GTA GCC GCT GAA ACT CC 3′ reverse 5′ TGT TCC CGC TCT CAT CTG C OTII: forward 5′ GCT GCT GCA CAG ACC TAC T 3′ reverse 5′ CAG CTC ACC TAA CAC GAG GA 3′ CRE: forward 5′ CGA TGC AAC GAG TGA TGA GG 3′ reverse 5′ GCA TTG CTG TCA CTT GGT CGT 3′

### Reagents

For flow cytometry, Fluor conjugated anti-CD3, CD4 and CD8 (Ebioscience) were used. For immunoblotting, anti-talin (8d4) and anti-actin antibodies (Sigma), anti-calpain 2 and anti-calpain 1 (Abcam), and anti-Rp3 calpain-s1 (Triple Point Biologics) were used. Recombinant proteins: CCL19, CCL21, SDF-1 alpha, rmICAM-1 were purchased from R and D systems. Fibronectin was purified as described [30].

### Cell Culture

Single cell suspensions of primary mouse T cells were made from lymph nodes and spleen from Capn4^+/+^:CD4-CRE and Capn4^F/F^:CD4-CRE mice that were between 12 and 16 weeks of age. Following red blood cell lysis, mixed lymphocyte populations were resuspended in complete RPMI supplemented with 25 u/ml IL-2 (Chiron) and stimulated with OVA_223–230_ (Anaspec). These OVApeptide expanded cells were used for in vitro assays days 7 to 10 following isolation for antigen-dependent experiments. Alternatively, CD4+ T cells were isolated from cell suspension by negative selection and automacs sorting (Miltenyi). Isolated CD4+ T cells were then stimulated 1∶1 with anti-CD3/CD28 coated beads according to manufacturer's instructions (Invitrogen) and maintained in RPMI supplemented with IL-2 (Chiron). These anti-CD3/CD28 bead activated cells were used for in vitro assays days 7 to 10 following isolation, unless otherwise noted. LB27.4 B cell were purchased from ATCC and maintained in RPMI complete media.

### Western Blotting

For immunoblotting, anti-CD3/CD28 bead activated CD4+ T cells were lysed in 50 mM Tris pH 7.6, 0.15 M NaCl, 0.1% SDS, 0.5% DOC, 1% NP-40 containing 0.2 mM PMSF, 1 ug/ml pepstatin, 2 ug/ml apoprotinin, 1 ug/ml apoprotinin, 1 ug/ml leupeptin and 1 mM sodium orthvanadate on ice and cleared by centrifugation. Protein concentration was determined by bicinchoninic acid protein assay kit (ThermoScientific). Equal concentrations of protein was added to SDS sample buffer, boiled and run on a 6–20% acrylamide gradient gel. Proteins were transferred to a nitrocellulose membrane and stained. Blots were imaged with an Odyssey Infrared Imaging System (Licor Biotechnologies).

### Casein Zymography

Casein zymography was performed as described previously [31]. Briefly, 20 million anti-CD3/CD28 bead activated CD4+ T cells from Capn4^+/+^:CD4-CRE, Capn4^+/F^:CD4-CRE and Capn4^F/F^:CD4-CRE mice were lysed and run on a casein-acrylamide gel under non-reducing conditions. Reactivation of calpains resulted in casein degradation. Gel was stained by coomassie and casein degradation is seen as an area of clearing.

### Flow Cytometry

2 inguinal and 2 cervical lymph nodes and spleen were isolated from Capn4^+/+^:CD4-CRE and Capn4^F/F^:CD4-CRE mice at 5 months of age. Thymocytes were isolated from 6 week old mice. 1 million naïve cells from each fraction were stained with anti-CD3, CD4 and CD8 antibodies and analyzed on a FACS Caliber (BD Bioscience). Total number of CD4+ T cells was determined based on initial cell counts from each fraction.

### Live Imaging

Anti-CD3/CD28 bead activated CD4+ T cells from Capn4^+/+^:CD4-CRE and Capn4^F/F^:CD4-CRE mice were plated on a glass bottom plate coated with poly-l-lysine and 3 ug/ml rmICAM-1 in RPMI complete media. Random migration was examined with an epifluorescent microscope (Nikon) using a Coolsnap ES2 camera (Photometrics). One bright field image was acquired every 30 seconds for 30 minutes. Images were acquired using MetaMorph Imaging software (MDS Analytical Technologies) and cell tracking completed in ImageJ. Directionality, as measured by the ratio of net distance traveled (D) divided by total path length (T), was determined as previously described [32].

### Immunofluorescence

LB27.4 B cells were stained with 1 uM CMAC (Invitrogen) according to manufacturer's directions and pulsed with 2.5 ug/ml OVApeptide for 30 minutes at 37 C. Equal numbers of OVApeptide expanded T cells and B cells in RPMI were combined, centrifuged and incubated for 20 minutes prior to resuspension in PBS and pulse vortexing. Cells were allowed to adhere to poly-l-lysine (Sigma) coated coverslips for 5 minutes at 37 C prior to fixing with 3% paraformaldehyde (Electron Microscopsy Services) for 15 minutes at 25 C. Cells were permeabilized with 0.2% Triton X-100, blocked in goat serum and stained with anti-PKC-theta (Santa Cruz), anti-LFA-1 (M17/4) (Ebioscience), and rhodamine phallodin (Invitrogen) along with FITC/TRITC conjugated anti-rat or FITC conjugated anti-rabbit secondary antibodies (Jackson Labs). Images were acquired on a laser scanning confocal microscope (Olympus) using a 60× Plan Apo/1.45 oil immersion objective with a 10× zoom factor and captured into Fluoview software (FV10-ASW version 01.07; Olympus).

### Transwell Chemotaxis

Transwell chemotaxis assays were performed as described with minor modifications [33]. Briefly, 3 um transwell chambers (Corning) were coated for 1 hour with 10 ug/ml fibronectin and blocked with 1% BSA. 100,000 anti-CD3/CD28 bead activated CD4+ T cells were added to the upper chamber and media alone, 100 ng/ml CCL19, CCL21 or SDF-1 alpha was added to the bottom chamber. Following a 6 hour incubation at 37 C, cells were removed from the filter by addition of EDTA and counted by flow cytometry. Percentage of CD4+ T cells transmigrated relative to a loading control was determined.

### Adhesion

Adhesion assays were performed as previously described [4]. Briefly, 96 well plates were coated with 3 ug/ml mICAM-1 (R and D systems) and blocked with 1% BSA. Anti-CD3/CD28 bead activated CD4+ T cells were stained with 0.5 ug/ml Calcein AM (Invitrogen) and left untreated or stimulated with 0.5 ug/ml biotinylated anti-CD3 (Ebioscience) and streptavidin (Jackson labs), 20 ng/ml PMA (Sigma) or 10 uM MnCl2 (Sigma). Cells were allowed to adhere to plates for 25 minutes at 37 C and a prewash fluorescence emission measured on a VictorV3 plate reader (Perkin Elmer). Plate was washed by pipetting and post-wash fluorescence emission measured. Percent adhesion determined by Fluorescence_initial_−Fluorescence _final_/Fluorescence_initial_.

### Conjugation

LB27.4 B cells were stained with 2.5 uM PKH-26 (Sigma) in 5% dextrose according to manufacturer's instructions and left untreated or loaded with 2.5 ug/ml OVApeptide for 30 minutes at 37 C. OVApeptide expanded CD4+ T cells were stained with 0.5 ug/ml Calcein AM (Invitrogen). Both cell types were resuspended in Hank's Buffered Salt Solution (Mediatech) supplemented with 2 mg/ml BSA (Sigma) and 1 mM Hepes (Mediatech). Equal numbers were combined on ice and centrifuged at 0.6 RCF for 5 minutes. Pellets were incubated at 37 C for indicated times prior to dissociation of non-specific conjugates by vortexing. Conjugates were analyzed using a FACS Caliber (BD Bioscience) and the percent conjugate formation determined by the percent double positive cells divided by the sum of the percent double positive and single positives.

### Proliferation

OVApeptide expanded CD4+ T cells were stained with 0.25 uM CFSE (Invitrogen) according to manufacturer's directions 10 days following isolation. Cells were left unstimulated or stimulated with one anti-CD3/CD28 coated bead (Invitrogen) per cell. Additionally, CD4+ T cells were stimulated with irradiated splenocytes (3000 gy) loaded with 0, 0.1 or 1 ug/ml OVA peptide. 72 hours following activation, cells were stained with anti-CD4 and dye dilution in CD4+ T cells analyzed using a FACS Caliber (BD Bioscience). Histograms were produced in FlowJo (Tree Star) and percentage of cells per division was determined using ModFit 3.2.1 (Verity) analysis program.

### Apoptosis

Apoptosis assays were performed as described in [34]. CD4+ T cells from Capn4^+/+^:CD4-CRE and Capn4^F/F^:CD4-CRE mice were stimulated with anti-CD3/CD28 coated beads for 4 days prior to treatment with either extrinsic or intrinsic apoptotic stimuli. Extrinsic stimuli included 15 ug/ml his-tagged FasL and 5 ug/ml anti-His antibody (R and D systems) and 100 ng/ml TNF alpha (R and D Systems). Intrinsic stimuli included 100 nM Etoposide (Sigma), 100 nM dexamethasone (Sigma) and 0.5 ug/ml biotinylated anti-CD3 (2C11) (Ebioscience) plus 1 ug/ml streptavidin (Jackson Labs). 30 hours following stimulation, T cells were stained with annexin V and propidium iodide according to manufacturer's directions (BD Bioscience) and the percentage of live cells determined by flow cytometry.

### Statistical Analyses

Statistical analyses were performed using Prism 4 software (GraphPad Software Inc). Two tailed paired T-test or one-way analysis of variance (ANOVA) was used with P<0.05 considered statistically significant. There were no statistically significant differences observed in any study.
